# Imidacloprid soil movement under micro-sprinkler irrigation and soil-drench applications to control Asian citrus psyllid (ACP) and citrus leafminer (CLM)

**DOI:** 10.1371/journal.pone.0192668

**Published:** 2018-03-08

**Authors:** Evelyn Fletcher, Kelly T. Morgan, Jawwad A. Qureshi, Jorge A. Leiva, Peter Nkedi-Kizza

**Affiliations:** 1 University of Florida (UF), Institute of Food and Agricultural Sciences (UF-IFAS), Putnam County Extension Office, East Palatka, Florida, United States of America; 2 UF-IFAS Southwest Florida Research and Education Center, Immokalee, Florida, United States of America; 3 UF-IFAS Indian River Research and Education Center, Fort Pierce, Florida, United States of America; 4 UF-IFAS Soil and Water Science Department, Gainesville, Florida, United States of America; Chinese Academy of Agricultural Sciences Institute of Plant Protection, CHINA

## Abstract

Imidacloprid (IM) is used to control the Asian Citrus Psyllid (ACP) and citrus leafminer (CLM), which are related to the spread of huanglongbing (HLB or citrus greening) and citrus canker diseases, respectively. In Florida citrus, imidacloprid is mainly soil-drenched around the trees for proper root uptake and translocation into plant canopy to impact ACP and CLM. The objective of this study was to determine the effect of imidacloprid rate, and irrigate amount on concentration of imidacloprid in the soil following drench application to citrus trees in three age classes. The plots were established at the Southwest Florida Research and Education Center, Immokalee, using a randomized complete-block design for three age classes of trees: one-year-old trees (B1), three to five-year-old trees (B2), and eight-year-old trees (B3). The treatments were a combination of two rates each of imidacloprid (1D, 2D) and micro-sprinkling irrigation (1I, 2I). Imidacloprid and bromide (Br^-^) used as tracer were applied simultaneously. Soil moisture and concentrations of imidacloprid and Br were monitored using soil cores from hand held augers. Soil moisture content (*θ*_*V*_) did not differ under two irrigation rates at any given observation day or depth, except following heavy rainfall events. Br^-^ was lost from the observation depths (0–45 cm) about two weeks after soil-drench. Contrarily, imidacloprid persisted for a much longer time (4–8 weeks) at all soil depths, regardless of treatment combinations. The higher retardation of imidacloprid was related to the predominantly unsaturated conditions of the soil (which in turn reduced soil hydraulic conductivities by orders of magnitude), the imidacloprid sorption on soil organic matter, and the citrus root uptake.

Findings of this study are important for citrus growers coping with the citrus greening and citrus canker diseases because they suggest that imidacloprid soil drenches can still be an effective control measure of ACP and CLM, and the potential for imidacloprid leaching to groundwater is minimal.

## Introduction

Imidacloprid (IM) is a systemic insecticide commonly used in home lawns, gardens, pets, and many agricultural commodities such as citrus, tomatoes, grapes, potatoes, and lettuce, just to name a few [1]. In Florida, imidacloprid use in citrus is common for the control of Asian citrus psyllid (ACP) which vectors Candidatus Liberibacter asiaticus (CLas) putative pathogens of “huanglongbing” (HLB) or citrus greening disease, as well as citrus leafminer (CLM) Phyllocnistis citrella Stainton (Lepidoptera: Gracillariidae) which exacerbates the spread of citrus canker. [2,3,4]. HLB is the most devastating disease of citrus [4,5]. HLB originated in China [5,6], and has spread to nearly all citrus producing areas of the world including the United States, South America, Central America, South Africa, South Korea, and Brazil [7]. The disease was first confirmed in Florida’s Miami-Dade County in 2005, and has spread as far north as Putnam County at the northern edge of the Florida citrus industry. By February of 2009, the disease had spread to 33 out of 64 Florida counties including nearly all commercial citrus production areas [8].

Imidacloprid is a neonicotinoid - a synthetic derivative of nicotine - and in Florida citrus it is most commonly applied on young, nonbearing citrus trees [9]. In Florida citrus, imidacloprid is applied as soil-drench using an applicator metered to deliver 8–10 oz of formulated solution per tree at the soil-rootstock interface [9]. Then, the irrigation system is activated to allow the active ingredient’s infiltration into soil to a depth of about 5 to 10 cm. Imidacloprid blocks the neural pathway in arthropods by stimulating the nicotinic acetylcholine receptors in the nervous system [10], which makes it effective against insects and less toxic to fish or mammals [11]. Imidacloprid acts systemically when soil-applied to plant roots, as it travels up the xylem and throughout the plant to tissues such as the leaves and pollen [12, 13]. Its effectiveness against ACP may last for 11 weeks after application [14]. The soil drench is conducted because citrus trees have shallow root systems that are concentrated within the first 90 cm of topsoil in the Florida Central Ridge soils (Entisols), and within 45 cm of topsoil in the southwest Florida flatwoods soils such as Alfisols, Spodosols, and Entisols [15]. Moreover, about 75% of the citrus feeder roots are present in the first 30 cm of soil [16]. When *C*Las infects the tree, the root biomass become less dense and more susceptible to diseases inhibiting water and nutrients uptake and further weaken the tree [17].

Imidacloprid data on soil sorption coefficients normalized to the soil organic carbon content (*K*_*OC*_) range from 156 to 960, and its half-life (t_1/2_) extends from weeks to months [18,19]. Laboratory studies on imidacloprid soil sorption and degradation conducted by Leiva et al. [20], as well as miscible displacement experiments have shown that imidacloprid is a weakly-sorbed (K_OC_ range 163–230) and persistent chemical (t_1/2_ range 1.0–2.6 years) during transport in plants in sandy flatwoods soils of Florida [21]. Moreover, evidence of imidacloprid leaching to groundwater at μg L^-1^ concentrations have been found in wells located at the Florida Central Ridge [22,23]. In general, soil-drenched systemic pesticides need to be evaluated in the field according to formulation, application rates, and irrigation management. For instance, imidacloprid has been found to have a positive relationship between application dosage and its stream concentrations in Appalachian managed forests [24]. Also, imidacloprid tablet formulations (rather than wettable powder) was found to improve uptake and control on the hemlock woolly adelgid and reduced leaching potential [25]. Drip-chemigation under plastic-mulch have also shown a significant reduction on the leaching potential of imidacloprid at the field level [26]. Alva et al. study in Candler fine sand showed the importance of the proper placement of fertilizers and pesticide drench in citrus groves [27]. They found higher water fluxes (and therefore higher leaching potential) below the citrus dripline of old-trees compare to positions below the citrus canopy. More recently, both micro-sprinkling and drip irrigation have been shown to have a positive effect on water and nutrient uptake in citrus [28,29] and could be a potential management strategy to enhance imidacloprid efficiency after the soil-drench application.

Imidacloprid is an effective systemic insecticide for young non-bearing citrus trees. Nonetheless, HLB affects citrus trees of all ages so it is important to estimate imidacloprid soil concentrations as a function of time after application, and its availability for uptake while monitoring and controlling ACP populations. Another key aspect of citrus (and imidacloprid) management is that little is known about the effects of irrigation rates on the potential leaching of soil-drenched organic chemicals. Therefore, the main objective of this study was to measure imidacloprid concentrations as a function of time after the drench application to increasingly larger soil areas (as citrus tree canopies increase in size with age) in experimental citrus groves on Immokalee fine sand. The study analyzed the effect of drench rates and micro-sprinkler irrigation on the overall field leaching pattern of imidacloprid in sandy profiles. This study was conducted during summer (rainy season) and spring (dry season) and also generated data on plant tissue concentrations of imidacloprid and ACP control under different drench and irrigation rates that will be discussed in a separate paper.

We believe that information on imidacloprid fate in soil, as a function of time after soil-drench application, will be useful for citrus managers and growers in Florida and other regions to improve control of the HLB vector using most effective and environmentally conscious approach. Our hypothesis was that imidacloprid would persist longer in soil when citrus trees are subjected to lower irrigation rates and higher imidacloprid concentration during drench application, without changing the frequency of irrigation. Another hypothesis was that, regardless of the initial imidacloprid soil concentration immediately after application, keeping soil moisture content below field capacity would increase imidacloprid retardation and therefore its persistence time in the citrus root zone. The longer soil persistence time would enhance imidacloprid uptake by the citrus trees and control of both ACP and CLM.

## Materials and methods

### Location and soils

No permit or specific permission was required, because these studies did not involve endangered or protected species. This study was conducted at the University of Florida, Institute of Food and Agricultural Sciences (UF-IFAS) Southwest Florida Research and Education Center (SWFREC), Immokalee (Lat. 26° 27.75’ N; Long. 81° 26.83’ W), where the dominant soil series is Immokalee fine sand (IFS), which is a flatwoods Spodosol commonly found in south Florida [30]. This soil series was formed from marine sedimentation, with slopes generally in the 0 to 2% range. The study soil taxonomy is sandy, siliceous, hyperthermic, Arenic Alaquods, and a typical profile contains the following master horizons: A, E1, E2, Bh1, Bh2, and BC. This soil is predominantly composed of deep layers of uncoated sand (E horizon) to a depth of approximately one meter, followed by a spodic layer or Bh horizon. During the rainy season (summer), the water table can be as high as 15 cm below the soil surface, and as deep as 150 cm during the dry season (spring). IFS properties in our experimental blocks are summarized in Table 1. The soil has sandy texture and low contents of both clay and organic carbon.

**Table 1:** Selected soil physical and chemical properties in experimental blocks (B1, B2, B3).

Block	Depth (cm)	pH ^c^	Texture (%)			SOC ^d^ (%)	Ksat ^e^ (cm h^−1^)	Bulk density (g cm^−3^)
			Sand	Silt	Clay			
B1 ^a^	0–15	5.5	93.8	5.0	1.2	0.80	3.6	1.55
B1	15–30	5.8	97.2	2.7	0.1	0.19	1.2	1.64
B1	30–45	5.8	98.4	0.5	1.1	0.10	0.8	1.68
B2 & B3 ^b^	0–15	5.6	98.0	1.2	0.8	0.30	16	1.55
B2 & B3	15–30	5.2	97.2	2.7	0.1	0.20	14	1.58
B2 & B3	30–45	5.8	95.0	2.5	2.5	0.24	13	1.55

^a^ Data from Leiva et al. [15].

^b^ Data from Kadyampakeni et al. [28].

^c^ pH in water, soil:solution ratio of 1:2.

^d^ Soil organic carbon.

^e^ Saturated hydraulic conductivity

### Experimental design and sampling

Experimental plots of ‘Hamlin’ orange trees (*Citrus sinensis* (L.) Osbeck) were established at the SWFREC previously, as part of a larger plot for citrus research. The study was conducted in a randomized complete-block design with 3 replications. Each block consisted of twelve trees of the same age: one-year-old trees (B1), three to five-year-old trees (B2), and eight-year-old trees (B3). The treatments in each block had a factorial design: two drench rates of imidacloprid (factor 1) and two micro-sprinkling irrigation rates (factor 2). Each treatment combination was applied to three trees that were planted next to each other. The average planting distance between trees and between rows (respectively) for B1 was 2.45 m by 5.50 m, for B2 was 3.05 m by 6.90 m, and for B3 was 4.50 m by 6.70 m. We measured the following variables at three soil depths (0–15, 15–30, 30–45 cm), and as a function of time after the drench application: soil moisture content (cm^3^ cm^-3^), imidacloprid soil concentration (μg cm^-3^), and the tracer (Br^-^) soil concentrations (μg cm^-3^).

**Fig 1:**
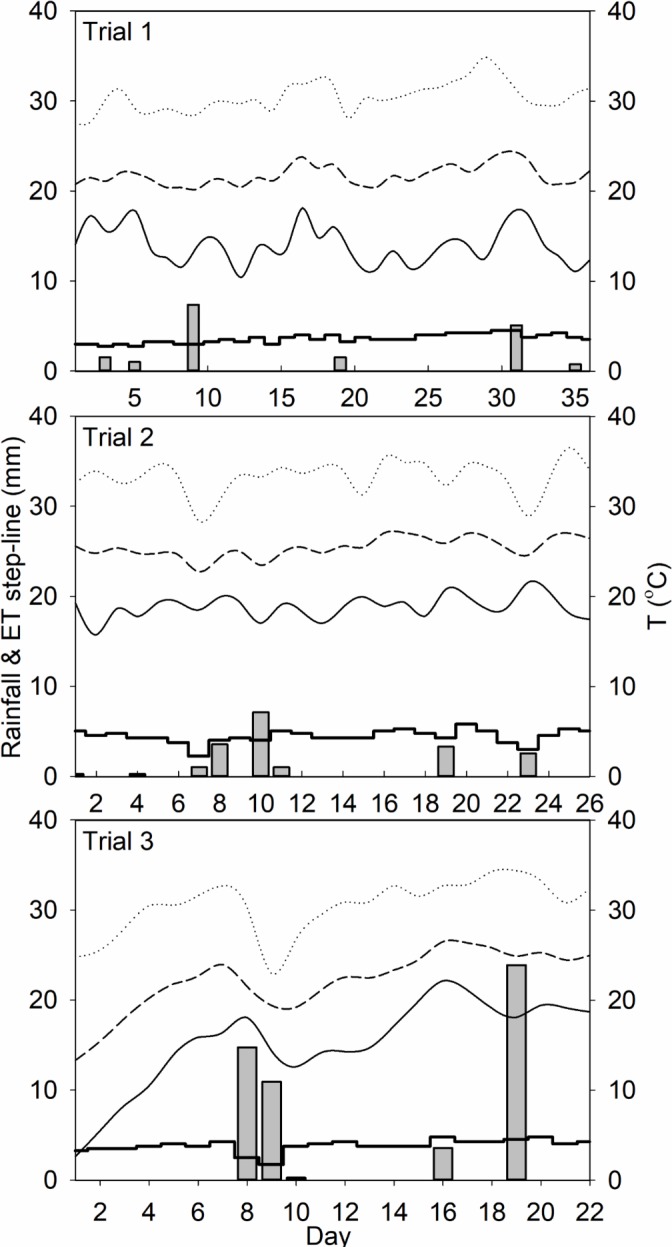
Rainfall (bars), evapotranspiration or ET (step-line), and temperature data (⋯ maximum, ₋ ₋ ₋ average, ― minimum) during trial 1 (Day 1: March 7^th^, 2012), trial 2 (Day 1: May 10^th^, 2012), and trial 3 (Day 1: March 28^th^, 2013).

The irrigation system consisted of micro-sprinkling emitters that were located alongside the tree-rows with each emitter located in between every other tree. On average, irrigation timing or frequency was 1.5 to 2 hours of irrigation, three times per week (Mondays, Wednesdays, Fridays), which was adjusted depending on climatic conditions. Data in Fig 1 shows precipitation and evapotranspiration [31] during our field experiments. Each micro-sprinkling emitter had a circular area of 2.6 m in diameter that covered two trees in the planting line. Irrigation water was applied at two different rates: 23 and 38 L h^-1^ (treatments 1I and 2I, respectively). Each combination of irrigation (1I and 2I) and drench (1D and 2D) rates was replicated on three trees, for a total of twelve trees per block (tree age class). All treatment combinations were applied to each block (and the same individual trees) during three trials corresponding to spring 2012, summer 2012 and spring 2013 growing seasons.

Background concentrations of imidacloprid in soils were measured to determine any imidacloprid residues prior to our soil-drench applications, along with the initial soil moisture content. Imidacloprid was soil-drenched by applying 250 mL of application solution within the drip line of each tree using a motorized sprayer in B1 for all three trials. After application, the trees were irrigated for 2 hours, as suggested by the label. In the second and third trials (summer 2012 and spring 2013), the drench application volume per tree was adjusted to 750 mL (using the same concentration) to apply a larger mass of active ingredient and account for the larger root biomass and tree canopy cover in B2 and B3. Imidacloprid soil-drench rates were based on label recommended concentrations for Admire-Pro Systemic Protectant [32], which is a concentrated liquid solution with 550 g L^-1^ of active ingredient. The label recommended a maximum application per year between 508 and 1015 mL ha^-1^. Since, the total rate is normally split into several applications per year, we determined that summer applications would have to be more concentrated because of the more intense rainfall and higher activity of the ACP during the summer growing season. The application rates (1D & 2D) for Trial 1 were 254 and 508 mL ha^-1^, in March and April of 2012. Trial 2, 1D and 2D rates were 508 and 1015 mL ha^-1^, in May and June of 2012. Trial 3 was conducted in spring 2013 (March-April) using the same drench rates for Trial 2.

The application tanks were also spiked with reagent grade NaBr (Sigma Aldrich) as a source of Br^-^ to use as tracer for water movement during the experiment, since it is a soluble anion not conspicuously present in IFS. We used a concentration that incorporated approximately one gram of Br^-^ per tree at soil-drenching. Therefore, each block of trees (B1, B2, B3) received a 2 x 2 combination of treatments (drench rate and irrigation rate): 1D+1I (Treatment 1), 1D+2I (Treatment 2); 2D+1I (Treatment 3); 2D+2I (Treatment 4). The Br^-^ was only applied in the 1D IM combinations.

### Soil sampling and extractions

Soil samples were collected at three depths (0–15, 15–30, and 30–45 cm) from several locations within the tree canopy using a stainless-steel bucket auger (15 cm length by 5.08 cm i.d.). The B1 soil samples were taken using a stainless-steel push probe (1.27 cm i.d.) to prevent disrupting the roots of these small non-bearing trees. During sampling, care was taken to clean the augers with nanopure-deionized water before sampling each location and depth. Soil samples were stored in lined sample bags (Fisherbrand), transported in coolers and immediately frozen at the field laboratory until extraction and analysis. The soil bulk density (g cm^-3^) was measured with stainless-steel cores, and was used to convert from gravimetric (g g^-1^) to volumetric measurements of water content (cm^3^ cm^-3^), Br^-^ (g cm^-3^) and IM (g cm^-3^).

Soil samples for imidacloprid extraction and analysis were thawed overnight at 4.4°C in a refrigerator. Soil water content was determined by taking subsamples from each bag and drying them in aluminum pans at 105°C for 24 hours. Then, 20 g of moist soil sample were weighed in a 50 mL polypropylene centrifuge tube, and 20 mL of extracting solution of methanol and 0.01M CaCl_2_ (80:20) was added. The tubes were shaken for two hours [33] and left to stand on laboratory benches for two hours for the soil particles to settle down. Centrifugation was performed at 6000 rpm for 15 minutes if required. Samples were then filtered using Whatman 42 filter paper into 20 mL scintillation vials. The extracts for imidacloprid were frozen at -12.2°C until HPLC-UV analysis.

Br^-^ soil samples were thawed in a refrigerator overnight before extraction. Soil moisture content was measured as previously indicated. Then, 20 g of soil sample were weighed in a 50 mL polypropylene centrifuge tube, and 20 mL of HPLC grade water was added. The tubes were shaken for 5 min at high speed in a reciprocating shaker. The tubes were left to stand for two hours, and centrifuged at 6000 rpm for 15 minutes if needed. The supernatant extract was filtered using Whatman 42 filter papers into 20 mL scintillation vials. Each vial contained between 10 to 20 mL of extract and were stored in the refrigerator at 4.4°C until analysis.

### Analytical methods

Soil imidacloprid extracts were analyzed at the UF-IFAS Environmental Soil Physics Laboratory, Soil and Water Sciences Department, Gainesville. The chromatographic conditions were based on previous analytical studies of imidacloprid in soil and water matrixes using HPLC-UV [33,34]. Analytical-grade standards for imidacloprid were obtained from ChemService, Inc. (West Chester, PA). A 100 μg mL^-1^ standard stock solution was prepared in methanol. Before each analysis, a matrix-matched calibration was built with the following levels 0, 0.01, 0.05, 0.1, 0.5, 1.0, 10.0 and 15.0 μg mL^-1^ in 40:60 (methanol:0.01 M CaCl_2_) using a serial dilution technique from the imidacloprid stock. The method detection limit was 0.01 μg mL^-1^. The extracts were analyzed using an Infiniti-1260 HPLC-UV system (Agilent Technologies, Hamburg), with a Supelcosil™ LC-18 (15 x 4.6 cm) column. After filtration, an aliquot of 2 mL of extract was transferred to HPLC vials for analysis. The injection volume was 30 μL, and the flow rate was 1.0 mL min^-1^. The mobile phase consists of 60% HPLC grade water and 40% methanol. The detection wavelength was set at 272 nm. Imidacloprid showed a symmetric peak with an average retention time of 3.8 min.

Br^-^ was analyzed at the UF-IFAS SWFREC, using a QuikChem® method (Lachat Instruments, method 30-135-21-1-A). The method is colorimetric and was performed in a flow-through auto-analyzer. Br^-^ in the extract was oxidized to bromine by Chloramine-T, and then bromine is substituted on phenol red to produce bromophenol blue [35–37]. Sodium thiosulfate was added to reduce Cl^-^ interference. The absorbance is measured continuously at 590 nm. The calibration range (0.1 to 10 μg Br^-^ mL^-1^) was linear, and the method detection limit was 5 ng Br^-^ mL^-1^.

### Statistical analysis

The data was analyzed using SigmaPlot 13.0 (SYSTAT, San Jose, California) using a nonparametric procedure: the Kruskal-Wallis (KW) analysis of variance on ranks. The IM volumetric concentrations, as well as the log10 and squared-root transforms showed non-normality (right skewed, and unequal variances), according to Shapiro-Wilk’s procedure. There were two combined factors: (i) drench rate and (ii) irrigation rate. Our goal was to estimate the effect of the resulting four combinations of soil-drench (1D, 2D) and irrigation (1I, 2I) on imidacloprid volumetric concentrations at different depths and sampling times after the drench application. The KW test was conducted for each sampling day by pooling all blocks and ranking all imidacloprid concentrations for each observation depth. When the KW test detected significant differences between ranked concentrations of imidacloprid, pairwise multiple comparisons were conducted using the Tukey procedure.

## Results

### Soil moisture and irrigation rates

**Fig 2:**
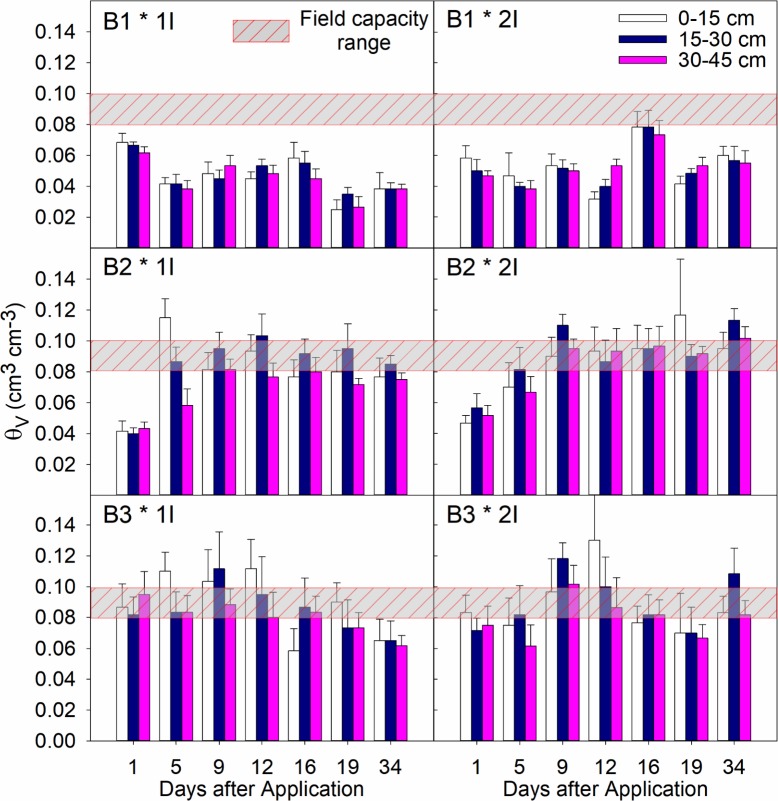
Soil moisture content (θ_v_, cm^3^ cm^-3^) at selected sampling days after soil-drench application during spring 2012 for trial 1 (Day 1: March 7^th^, 2012). Grouped-vertical bars represent the 3 sampling depths (0–15, 15–30, 30–45 cm), with averages and standard error bars (n = 6). The horizontal bars represent the average field capacity for Immokalee fine sand. B1: one-year-old trees; B2: 3 to 5-year-old trees; and B3: 8-year-old trees. Irrigation treatments: 1I = 23 L h^-1^; 2I = 38 L h^-1^.

**Fig 3:**
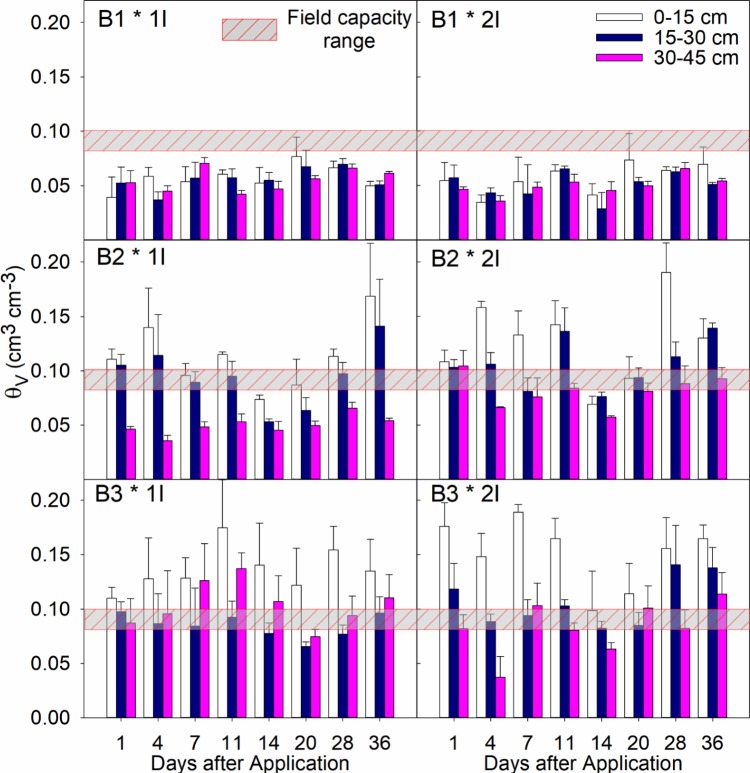
Soil moisture content (θ_v_, cm^3^ cm^-3^) at selected sampling days after soil-drench application during summer 2012 or trial 2 (Day 1: May 10^th^, 2012). Grouped-vertical bars represent the 3 sampling depths (0–15, 15–30, 30–45 cm), with averages and standard error bars (n = 6). The horizontal bars represent the average field capacity for Immokalee fine sand. B1: one-year-old trees; B2: 3 to 5-years-old trees; and B3: 8-years-old trees. Irrigation treatments: 1I = 23 L h^-1^; 2I = 38 L h^-1^.

Averages and standard errors (n = 6) for any given sampling day or depth were calculated based on irrigation rate, without considering drench application rates (1D & 2D) for trial 1 and trial 2 (spring 2012 and summer 2012) in Figs 2 and 3, respectively. In general, the soil moisture content (θ_V_) showed no important differences between the two irrigation treatments at any given day or depth. For instance, during Trial 1 in B1-1I data (young trees, 23 L h^-1^) the soil samples had an average θ_V_ of 0.07 in the 0–15 cm depth, 0.07 in the 15–30 cm depth, and 0.06 in the 30–45 cm depth. For the same group of trees (B1), the 2I irrigation treatment (38 L h^-1^) had an average θ_v_ of 0.05 for all depths. The same trend was observed for the blocks of 3–5 years-old trees (B2) and the 6–8 years-old trees (B3).

Also, sampling was conducted several hours after irrigation events, allowing redistribution of infiltration water between the observation depths. The only exception to this trend was the evident spikes in θ_V_ for the 0–15 and 15–30 cm sampling depths observed during summer season or Trial 2 (Fig 2, panels B2 and B3), because of heavy rainfall events. Regardless of irrigation treatment, θ_V_ was greater for the 0–15 and 15–30 cm depths, at most sampling times where the moisture content was significantly higher than the field capacity for IFS, which is estimated to be around a θ_V_ value of 0.10 cm^3^ cm^-3^ [38]. During our experiments, there was no evidence of saturation (θv ≈ 0.38 cm^3^ cm^-3^). Also, average θ_V_ for block B1 (young non-bearing citrus trees) was significantly lower than blocks B2 and B3, at any given day or sampling depth. This reduction in soil moisture content was evident during all sampling seasons and is typically because of increased surface evaporation.

### Tracer concentrations after soil-drench

**Fig 4:**
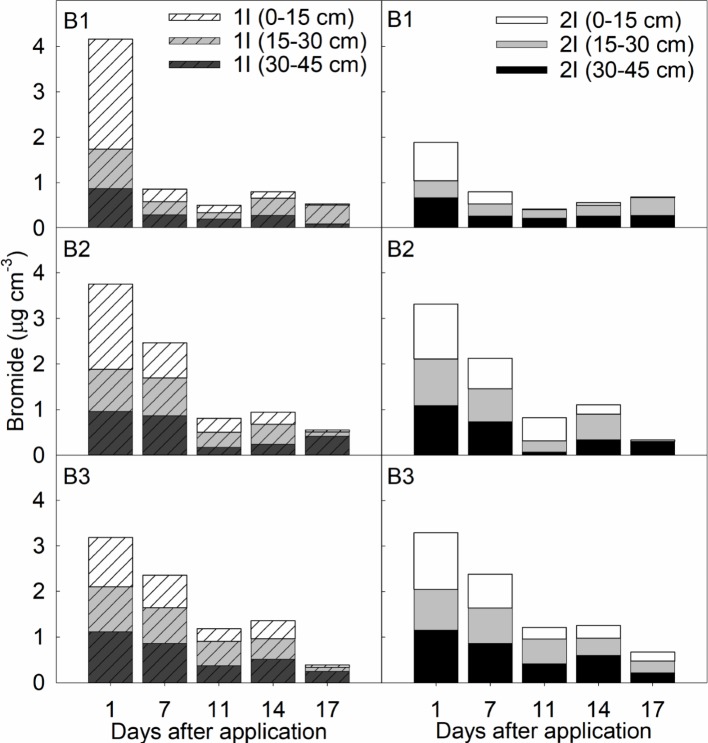
Bromide concentrations detected (μg cm^-3^) after soil-drench application during trial 3 (spring 2013) at sampling depths 0–15 cm (white bars), 15–30 cm (gray bars), and 30–45 cm (black bars) in 1 year-old trees (B1), 3–5 year-old trees (B2), and 8 year-old trees (B3).

Tracer concentration from Trial 3 (Fig 4) explained the overall trend for the tracer during all experimental trials conducted, which we assumed represent the wetting front after application. Except for the data in B1 at one day after application (DAA), initial concentrations showed no differences in Br^-^ concentrations between irrigation rates 1I and 2I regardless of sampling time and depth. Less than 0.1 μg Br^-^ cm^3^ was observed at 0–15 cm 14 DAA, and less than 0.3 μg Br^-^ cm^2^ remained in the 15–30 cm and 30–45 cm depths. In the youngest trees (B1), about 13% of Br^-^ applied remained in the soil 17 DAA in the 23 L h^-1^ irrigation rate (1I), while about 35% remains with 38 L h^-1^ irrigation rate (2I). In the 3-5-year-old trees (B2), 14% of Br^-^ remained in the 23 L h^-1^ irrigated trees, and 10% remains in the 38 L h^-1^ irrigated trees. The oldest trees at 8 years-old (B3) showed 12.5% of Br^-^ applied remaining 17 DAA with 23 L h^-1^ irrigation, while 20% remained in the higher irrigation rate. Br^-^ leached out of the 0–15 cm depth and increased in concentration in the lower depths, particularly in 15–30 cm. There were no traces of Br^-^ at the soil surface (0–15 cm) by 11 DAA in the 38 L h^-1^ irrigation treatment, or by 17 DAA with the 23 L h^-1^ irrigation treatment in the youngest trees (B1). Therefore, the wetting front of the initial application pulse (250 or 750 mL per tree) leached out of the 0–15 cm depth about two weeks after application.

### Imidacloprid concentrations after soil-drench

Imidacloprid was sorbed or retained by the soil matrix for a longer time compared to the Br^-^ tracer, which was attributed to the affinity or sorption onto the soil organic matter. This trend was true regardless of the initial drench application rate and irrigation treatment, and was consistent across sampling seasons. Imidacloprid soil concentrations for Trials 2 and 3 are shown in Tables 2 and 3, respectively. In general, imidacloprid concentrations were higher in the 2D treatments compared to 1D, especially during the first week after application. The results from the Shapiro-Wilk test showed that the data did not satisfy the normality features required to conduct parametric analysis of variance.

**Table 2:** Imidacloprid average concentration (ng cm^-3^; n = 3) and standard deviations (in parenthesis) at three depths during trial 2 (summer 2012) for two soil-drench application rates (1D, 2D) and two irrigation rates (1I, 2I) in blocks B1, B2, and B3. Kruskal-Wallis test results (for each day and depth) are included.

Block/Treatment	Depth (cm)	Day after application (DAA)						
		1	4	7	11	14	20	25
		**Mean Imidacloprid Concentration, ng cm**^**−3**^ **(standard deviation)**						
B1 1D-1I	0–15	1958 (392)	1102 (217)	331 (114)	1640 (135)	1207 (18)	1065 (249)	1065 (176)
	15–30	326 (28)	776 (74)	350 (179)	210 (23)	218 (41)	208 (22)	587 (369)
	30–45	365 (109)	237 (37)	235 (1)	201 (1)	199 (2)	209 (14)	222 (42)
B1 1D-2I	0–15	1638 (263)	1216 (580)	232 (13)	238 (89)	183 (3)	312 (117)	216 (55)
	15–30	287 (69)	315 (25)	240 (19)	197 (1)	202 (9)	250 (23)	143 (43)
	30–45	363 (82)	223 (40)	235 (1)	201 (1)	201 (3)	201 (1)	198 (1)
B1 2D-1I	0–15	3288 (876)	1330 (70)	1560 (87)	1965 (130)	1660 (155)	2030 (155)	1026 (260)
	15–30	343 (28)	307 (26)	396 (116)	332 (89)	249 (36)	224 (48)	299 (129)
	30–45	295 (28)	216 (30)	270 (38)	291 (83)	225 (42)	254 (44)	331 (2)
B1 2D-2I	0–15	5523 (2639)	1491 (308)	709 (280)	665 (50)	581 (231)	442 (113)	302 (23)
	15–30	539 (176)	245 (50)	242 (21)	197 (2)	196 (1)	211 (16)	248 (19)
	30–45	678 (277)	200 (1)	281 (75)	225 (22)	200 (2)	212 (20)	212 (23)
B2 1D-1I	0–15	353 (46)	291 (87)	260 (59)	219 (31)	221 (46)	193 (9)	572 (227)
	15–30	246 (21)	201 (21)	249 (90)	231 (71)	189 (1)	229 (65)	433 (32)
	30–45	262 (31)	191 (5)	195 (2)	187 (1)	186 (1)	185 (1)	179 (1)
B2 1D-2I	0–15	377 (87)	669 (87)	194 (13)	389 (36)	236 (46)	237 (83)	626 (87)
	15–30	285 (25)	198 (8)	198 (12)	200 (13)	191 (1)	192 (1)	495 (157)
	30–45	267 (7)	187 (1)	187 (8)	189 (1)	186 (1)	188 (2)	382 (50)
B2 2D-1I	0–15	1035 (222)	701 (19)	891 (258)	245 (53)	228 42)	324 (82)	527 (81)
	15–30	287 (11)	192 (2)	188 (8)	194 (2)	189 (1)	190 (2)	318 (116)
	30–45	264 (54)	194 (13)	232 (63)	186 (1)	185 (1)	191 (9)	194 (12)
B2 2D-2I	0–15	1062 (181)	197 (1)	196 (6)	264 (73)	211 (41)	244 (55)	594 (159)
	15–30	335 (44)	239 (75)	229 (37)	199 (4)	219 (25)	240 (82)	713 (443)
	30–45	243 (37)	186 (1)	227 (70)	187 (1)	185 (1)	186 (1)	371 (166)
B3 1D-1I	0–15	249 (50)	199 (10)	194 (4)	198 (9)	195 (8)	233 (52)	182 (2)
	15–30	349 (115)	232 (34)	195 (11)	199 (5)	191 (5)	191 (2)	185 (2)
	30–45	260 (48)	212 (37)	194 (7)	194 (3)	191 (4)	187 (1)	184 (5)
B3 1D-2I	0–15	423 (41)	197 (4)	201 (1)	198 (4)	203 (26)	254 (92)	188 (7)
	15–30	351 (32)	213 (20)	194 (2)	195 (1)	192 (1)	193 (2)	188 (5)
	30–45	227 (23)	185 (4)	188 (2)	191 (8)	189 (9)	186 (3)	182 (3)
B3 2D-1I	0–15	301 (154)	194 (20)	194 (8)	208 (23)	201 (20)	205 (18)	184 (2)
	15–30	264 (35)	204 (16)	193 (7)	194 (3)	192 (3)	190 (1)	191 (5)
	30–45	263 (89)	194 (15)	197 (23)	189 (4)	185 (2)	189 (7)	184 (4)
B3 2D-2I	0–15	491 (148)	324 (118)	269 (76)	191 (5)	215 (26)	197 (13)	184 (2)
	15–30	260 (72)	191 (6)	194 (6)	218 (35)	194 (3)	189 (3)	189 (4)
	30–45	256 (49)	219 (33)	217 (49)	188 (1)	186 (1)	190 (4)	185 (2)
		**Kruskal-Wallis Tests (H-statistic and corresponding P value)**						
Pooled data (n = 9; df = 3)	0–15	4.10 (0.25)	0.77 (0.86)	4.11 (0.25)	5.99 (0.11)	5.72 (0.13)	2.36 (0.50)	4.96 (0.18)
	15–30	2.03 (0.57)	2.57 (0.46)	2.90 (0.41)	9.65 (0.02)+	7.13 (0.07)	1.47 (0.69)	3.90 (0.27)
	30–45	0.96 (0.81)	0.57 (0.90)	2.45 (0.49)	0.76 (0.86)	0.90 (0.83)	0.76 (0.86)	5.57 (0.14)

1D = 508 mL ha^-1^, 2D = 1015 mL ha^-1^, 1I = 23 L h^-1^; 2I = 38 L h^-1^. df: degrees of freedom for the KW-H.

+ KW test was significant. The differences in the *median* values among the *treatments were* greater than would be expected by chance.

**Table 3:** Imidacloprid average concentration (ng cm^-3^; n = 3) and standard deviations (in parenthesis) at three depths during trial 3 (spring 2013) for two soil-drench application rates (1D, 2D) and two irrigation rates (1I, 2I) in blocks B1, B2, and B3. Kruskal-Wallis test results (for each day and depth) are included.

Block/Treatment	Depth (cm)	Days after application (DAA)					
		1	6	11	14	18	21
		**Mean Imidacloprid Concentration, ng cm**^**-3**^ **(standard deviation)**					
B1 1D-1I	0–15	1037 (434)	1033 (514)	987 (664)	492 (202)	900 (1)	372 (87)
	15–30	1244 (11)	172 (2)	310 (1)	446 (143)	501 (103)	453 (347)
	30–45	600 (120)	134 (139)	169 (1)	92 (10)	128 (51)	363 (271)
B1 1D-2I	0–15	328 (29)	695 (*)	499 (179)	397 (334)	511 (*)	471 (*)
	15–30	303 (268)	555 (75)	243 (*)	568 (*)	-	-
	30–45	77 (60)	413 (217)	187 (*)	84 (*)	-	187 (*)
B1 2D-1I	0–15	556 (70)	715 (15)	668 (64)	543 (106)	459 (150)	543 (*)
	15–30	380 (245)	480 (*)	465 (58)	64 (*)	146 (35)	232 (225)
	30–45	782 (228)	558 (178)	276 (88)	213 (68)	122 (45)	116 (35)
B1 2D-2I	0–15	820 (59)	580 (155)	290 (75)	-	-	49 (*)
	15–30	64 (27)	92 (7)	308 (203)	68 (*)	114 (*)	-
	30–45	114 (32)	-	204 (51)	-	-	-
B2 1D-1I	0–15	426 (117)	765 (457)	473 (158)	811 (327)	875 (405)	477 (*)
	15–30	88 (44)	209 (29)	-	280 (122)	195 (78)	164 (41)
	30–45	40 (27)	165 (141)	-	182 (99)	320 (*)	101 (71)
B2 1D-2I	0–15	419 (13)	625 (274)	261 (181)	305 (112)	411 (15)	293 (17)
	15–30	53 (9)	89 (74)	171 (53)	292 (101)	323 (226)	54 (31)
	30–45	35 (7)	184 (47)	102 (1)	149 (41)	110 (36)	71 (18)
B2 2D-1I	0–15	2217 (440)	1232 (44)	996 (77)	1418 (275)	1363 (76)	565 (136)
	15–30	225 (172)	523 (356)	302 (235)	494 (81)	641 (94)	519 (153)
	30–45	44 (10)	88 (32)	549 (233)	255 (44)	183 (58)	131 (26)
B2 2D-2I	0–15	1946 (166)	1229 (43)	896 (615)	646 (466)	677 (176)	480 (182)
	15–30	148 (70)	312 (131)	133 (69)	349 (299)	317 (135)	209 (102)
	30–45	93 (10)	204 (*)	92 (17)	270 (23)	237 (55)	160 (20)
B3 1D-1I	0–15	1117 (85)	1111 (83)	1042 (782)	853 (399)	773 (117)	103 (42)
	15–30	170 (161)	357 (42)	206 (*)	547 (23)	428 (88)	356 (5)
	30–45	48 (26)	194 (*)	93 (*)	-	-	279 (*)
B3 1D-2I	0–15	1287 (422)	449 (127)	385 (285)	525 (130)	190 (110)	188 (102)
	15–30	93 (79)	357 (42)	137 (62)	571 (59)	-	175 (145)
	30–45	218 (12)	179 (30)	164 (31)	338 (275)	107 (8)	143 (*)
B3 2D-1I	0–15	3344 (239)	1857 (266)	2085 (765)	1329 (163)	1279 (330)	155 (49)
	15–30	79 (66)	212 (93)	-	161 (*)	82 (*)	191 (*)
	30–45	394 (95)	73 (*)	275 (33)	328 (184)	106 (51)	400 (97)
B3 2D-2I	0–15	1010 (*)	1214 (77)	801 (28)	1684 (214)	1530 (366)	833 (723)
	15–30	170 (161)	360 (7)	180 (*)	209 (147)	145 (*)	280 (*)
	30–45	57 (28)	151 (63)	230 (157)	290 (157)	372 (85)	659 (64)
		**Kruskal-Wallis Tests (H-statistic and corresponding P value)**					
Pooled data (n = 9; df = 3)	0–15	11.2 (0.01)^+^	15.0 (0.002)^+^	12.5 (0.006)^+^	8.14 (0.04)^+^	13.3 (0.004)^+^	2.61 (0.46)
	15–30	3.36 (0.34)	1.36 (0.72)	2.43 (0.49)	6.35 (0.10)	10.1 (0.02)^+^	11.3 (0.01)^+^
	30–45	3.68 (0.30)	6.21 (0.10)	19.2 (0.001)^+^	8.28 (0.04)^+^	5.83 (0.12)	6.44 (0.09)

1D = 508 mL ha^-1^, 2D = 1015 mL ha^-1^, 1I = 23 L h^-1^; 2I = 38 L h^-1^. df: degrees of freedom for the KW-H.

- Soil concentration was below method limit of detection.

* Value reported was one-out-of-three replicates that were above the method limit of detection.

+ KW test was significant. The differences in the *median* values among the *treatments were* greater than would be expected by chance.

The lower imidacloprid concentrations observed in blocks B2 (3–5 years old tree) and B3 (8 years old tree) during trial 2 (Table 2) were attributed to the larger application area where imidacloprid was soil-drenched. Also, larger trees have a significantly higher transpiration (and water uptake) rates than smaller citrus trees, which would reduce imidacloprid concentration in soil during the growing season. This was one of the main reasons that imidacloprid was applied at higher rates during both trials 2 and 3 (Tables 2 and 3), to compensate for the larger canopy volume and transpiration stream. For instance, in Trial 3 block B3 showed the largest concentrations of imidacloprid at any given observation depth during our experiments.

During Trial 2 (summer 2012) differences in soil imidacloprid concentrations among the four treatment combinations (drench rates by irrigation rates) were not statistically important based upon the results of the KW test for most sampling times (Table 2, H statistics < 9.65, P values ranging from 0.07 to 0.90). The only exception was during Day 11 where the 15–30 cm data showed a significant difference between the 2D-1I and the 2D-2I treatments (Tukey’s q = 4.24, P = 0.014). Furthermore, the larger variability in imidacloprid data added considerable noise for detecting differences between combinations of drench rates (1D, 2D) and irrigation rates (1I, 2I). Neither log10- nor root-square-transformed data satisfied the required assumptions of data normality. Nevertheless, the data trend showed a higher affinity or sorption of imidacloprid to the soil organic matter, which increased soil residence time compared to the Br^-^ tracer. Also, imidacloprid soil concentrations were consistently higher in the first depth of observation (0–15 cm) during most of the sampling days.

Trial 3 (spring 2013) data and corresponding KW tests showed differences between the ranked values of imidacloprid concentrations (Table 3), specially for the 0–15 cm observation depth (Table 4). The Tukey procedure showed consistently higher concentrations in the 2D-1I treatment (highest drench rate and lowest irrigation rate) compared to the 1D-2I treatment. This trend was consistent for most sampling dates, except Day 21. The other pairwise comparisons were not statistically different between the treatments (q statistics and P values are not shown). It may be that the lower variation in soil moisture contents during spring time (see Fig 2) as well as the lower frequency of heavy rainfall events, yielded lower noise during soil sampling allowing a better detection of the treatment effects on imidacloprid soil concentrations.

**Table 4:** Imidacloprid median concentrations (ng cm^-3^; n = 9) at 0–15 cm depth during trial 3 and Tukey multiple comparison results.

Treatment combination	Days after drench application				
	Day 1	Day 6	Day 11	Day 14	Day 18
1D-1I	1020	1033	580	705	890
1D-2I	419 b	441 b	320 b	384 b	290 b
2D-1I	2217 a	1232 a	996 a	1150 a	1280 a
2D-2I	1780	1186	772	646	619
Tukey's q *	2.9	5.2	4.9	4	4.8
(P value)	(0.02)	(0.01)	(0.01)	(0.02)	(0.01)

1D = 508 mL ha^-1^, 2D = 1015 mL ha^-1^, 1I = 23 L h^-1^; 2I = 38 L h^-1^

* q statistic for the 1D-2I vs 2D-1I comparison.

## Discussion

### Unsaturated conditions during field trials

The similarity of θ_V_ between observation depths and irrigation rates were probably due to the procedures followed for sampling and the homogeneous particle-size distribution of these soils (Table 1). The coarse nature of IFS (sand fraction >94% w/w) and a porous fraction mostly composed of macropores facilitated water infiltration and reduced lateral redistribution or dispersivity [39]. It is also possible that older (and larger) citrus trees in experimental blocks B2 and B3 intercepted more rainfall and/or micro-sprinkler irrigation during our field experiments. This phenomenon has been documented by Alva et al. [27] when they found that a larger tree canopy was one of the main factors explaining higher soil water fluxes directly below the tree dripline, a condition that would increase moisture content in these blocks.

Moreover, common weed control practices in Florida citrus rely upon herbicide application around the tree planting line to keep the soil surface uncovered during most of the year. This condition would certainly increase evaporation and reduce moisture content at the soil surface in B1. Conversely, the B2 and B3 larger canopy cover reduced evaporation rates from the surface, which was evident in the higher soil moisture content at most observation days and depths. The second trial experienced higher values of soil moisture throughout the season due to the higher frequency of rainfall during summer. The experimental site received 69 mm of rain during the span of the second trial, while only 16 mm of rain occurred during the first trial (Fig 1). The θv for Trial 3 (data not shown) followed a similar trend as Trial 1, with the main difference in the larger rainfall events that increased θv to values larger than field capacity (<0.10 cm^3^ cm^-3^). Nonetheless, saturated conditions during our sampling (θv ≈ 0.38 cm^3^ cm^-3^) were never recorded.

Also, there were small differences in the average θv between the two irrigation rates (1I, 2I), at any given depth or sampling day during Trials 1, 2, and 3. This trend was mainly attributed to supplemental irrigation that replenished daily evapotranspiration (ET) without creating saturated conditions nor saturated flow, which would consequently create a drastic decline in the soil hydraulic conductivity. Using the model by van Genuchten, Leiva [21] showed that IFS hydraulic conductivities (*Kh*) would be several orders of magnitude lower (<0.01 cm day^-1^) than the *Kh* values at saturation (>100 cm day^-1^). Also, redistribution of soil water after micro-sprinkler irrigation events allowed the soil moisture content to remain relatively constant between irrigation treatments, or well below the field capacity value, which increases the retardation factor of an adsorbed solute like imidacloprid almost three times.

### Imidacloprid movement and retardation in Florida sandy soils

Previous research has shown that imidacloprid is sorbed by the soil organic matter of IFS [20], which has a very low content compared to other agricultural soils. Based on the convective-dispersive equation for water and solute transport, the prevalent conditions in our field experiments increased the retardation factors of imidacloprid compared to the Br^-^ tracer which has a retardation of 1 and leached out of the root zone about 2 weeks after application (Fig 4). On the contrary, imidacloprid was still present in the soil profile even four to six weeks after application (Tables 2 and 3). In this regard, the best available models for Imidacloprid transport in IFS - the convective-dispersive model (CD-model) and the one-site nonequilibrium model (OSNE) - were studied in detailed by Leiva et al. [40]. Shortly, both models describe the solute transport process, and the models’ dimensionless form have a variable called the Retardation Factor (*R*), which is a function of Imidacloprid soil sorption coefficient (*K*_*D*_, mL g^-1^) and the volumetric soil moisture (*θv*, in cm^3^ cm^-3^). According to the following equation *R = 1+[(K*_*D*_ *ρ*_*b*_*)/θ]*, *R* is inversely proportional to the soil moisture content *θv*. The bulk density - *ρ*_*b*_ - is expressed in g cm^-3^. Therefore, keeping the soil moisture content around or below the IFS field capacity value (*θv* < 10%) increased the *R* value for imidacloprid by a factor of three compared to IFS when saturated (θ≈38%). In essence, the residence time of Imidacloprid in IFS was at least three times longer than the tracer.

Moreover, trends in ACP mature and immature populations (data not shown) agreed with longer retention times of imidacloprid in these soils, because of significant ACP population reductions about two weeks after imidacloprid soil-drench, with ACP populations under sustained control in soil-drenched plots for 4-to-8 weeks.

Results of this study are important for citrus growers currently affected by citrus greening. Findings indicate that micro-sprinkler irrigation systems will keep the citrus rootzone unsaturated at most times, significantly reducing the chance for imidacloprid to be lost by leaching to groundwater, even in the sandy soils of Florida flatwoods. Moreover, imidacloprid has shown to be very persistent in Immokalee Fine Sand [20, 21]. This should help increase its uptake by the citrus roots and later availability in the plant tissues for effectiveness against target pests.
